# Localized co-delivery of CNTF and FK506 using a thermosensitive hydrogel for retina ganglion cells protection after traumatic optic nerve injury

**DOI:** 10.1080/10717544.2020.1748759

**Published:** 2020-04-30

**Authors:** Dongmei Wang, Mengmeng Luo, Baoshan Huang, Wa Gao, Yan Jiang, Qing Li, Kaihui Nan, Sen Lin

**Affiliations:** a School of Ophthalmology & Optometry and Eye Hospital, Wenzhou Medical University, Wenzhou, China;; c State Key Laboratory of Ophthalmology, Optometry and Visual Science, Wenzhou Medical University, Wenzhou, China;; b Wenzhou Institute, University of Chinese Academy of Science, Wenzhou, China

**Keywords:** Thermo-sensitive hydrogel, ciliary neurotrophic factor, FK506 micelle, retina ganglion cells, traumatic optic nerve injury

## Abstract

Following the traumatic axonal injury in the optic nerve, the failure of retrograde axonal transport to continuously supply neurotrophins from the brain to retina results in deprivation of neurotrophins in retinal ganglion cells (RGCs), which in turn can modulate the fate of RGCs toward apoptosis and thereby impede axon regeneration. In this study, a ciliary neurotrophic factor (CNTF) loaded thermo-sensitive hydrogel was designed and developed as a localized drug depot to restore neurotrophins supply following axon injury. Besides, following traumatic axon injury, overactive immune responses cause neurotoxicity and induce scar formation which together constitutes the major hindrances for axon regeneration. Thus, the FK506, a hydrophobic macrolide immunosuppressant, was co-loaded into the hydrogel after encapsulating it into a polymeric micelle. The materials can undergo sol-to-gel transition within minutes under a physiological pH of 37 °C. The release of drugs from the hydrogel exhibited a sustainable profile *in vitro*. The optic nerve was exposed by surgical procedure and the animal model was prepared by crushing the nerve with a reverse clamp. For the localized delivery to the optic nerve, a pre-hydrogel liquid containing chitosan, FK506 (in micelle), CNTF, and the gelling agent was directly smeared on the injured site, which gelled under physiological condition. This co-delivery system exhibited *in vivo* RGCs protective effect against the adverse effects caused by traumatic optic nerve injury, indicating the potential of this drug delivery system for effective optic nerve repair and this strategy may provide promising platforms for localized drug delivery in various other therapies.

## 1. Introduction

The optic nerve, a paired cranial nerve II, is the convergence of retinal ganglion cells (RGCs) axons at the optic disk. The RGCs axons exit the optic disk to the brain through over one million nerve fibers that carry visual signals from the retina to the vision centers of the brain via electrical impulses. Injury of the optic nerve caused by eye traumas (traumatic optic neuropathy; TON), or optic neuropathy including inflammatory disorder, glaucoma, ischemia, and tumor mass compression can cause severe loss of vision (Fischer & Leibinger, 2012; Shum et al., 2016). Besides, any insult to the optic nerve leads to disconnection of the RGCs and its axons to the rest of the brain, entailing that the visual signals captured by the retina are no longer carried to the brain. Apparently, the axon of the optic nerve has limited endogenous ability to regenerate due to complex bio-mechanism including the failure of mature RGCs to regenerate injured axons, which subsequently induces death of RGCs, whereas degeneration of RGCs leads to the formation of scar at the site of injury (Fischer & Leibinger, 2012). To date, no therapeutic method had been able to efficiently regenerate the optic nerve or able to functionally repair axonal connections following-TON in clinical practice. However, studies during the past two decades have indicated that combined treatment of *in situ* inhibition of scar formation at the injured site and stimulation of RGCs axon to transform into an active regenerative state might enable these neurons to survive and to regenerate axons, for the effective treatment of TON (Fischer & Leibinger, 2012). Predominantly, treatment to maintain RGCs viability post TON, which ensure the potential for RGCs axon re-generation remains the key for optic nerve repair since RGCs death occurs within a short period of damage to the axon (in 5–14 days depends on the distance of the injury site to eyeball) (Almasieh et al., 2012; Shum et al., 2016).

However, the complex molecular mechanism underlying RGCs death following TON remains currently largely unknown (Almasieh et al., 2012). Conceivably, retrograde axonal transport of neurotrophic factor, the neurotrophins, from the target site (superior colliculus) to neuron is believed to be essential for RGCs survival as well as maintenance of its biological function (Quigley et al., 2000; Reynolds et al., 2000). Following axonal damage or TON, an interruption between RGCs and their target in brain impairs the ability of retrograde axonal transport of neurotrophins, leading to deprivation of neurotrophins in neuron, which in turn can modulate the fate of RGCs toward apoptosis (Fischer & Leibinger, 2012; Shum et al., 2016; Berry et al., 2019). Indeed, exogenous supplementation with brain-derived neurotrophic factor (DBNF) (Weber & Harman, 2013), glia-derived neurotrophic factor (GNDF)(Jongen et al., 2005), and nerve growth factor (NGF)(Colafrancesco et al., 2011) or viral-mediated gene therapy to restore neurotrophins supply (Feng et al., 2017; Tronci et al., 2017) showed their protective effects on RGCs *in vivo* in animal trials. Nevertheless, neurotrophic factors (including BDNF), despite exhibiting RGCs protective effects following TON, do not promote RGCs axon re-growth (Fischer & Leibinger, 2012). Beside the RGCs protective effects, ciliary neurotrophic factor (CNTF) promotes neurite outgrowth of RGCs through activation of the JAK/STAT3-, PI3K/AKT- and MAPK/ERK-signaling pathways (Müller et al., 2009; Bei et al., 2016), indicating that CNTF is a potent axon growth-promoting factor for mature RGCs following TON. Furthermore, vitreous injection of recombined CNTF exhibited less significant axon re-growth promoting effect then the vial mediated in vivo produced CNTF (Fischer & Leibinger, 2012; Hellstrom et al., 2011), indicative a constant supply of CNTF are needed for effective neuroprotection and axon re-growth after TON. Besides, overactivation of immune response occurs both in acute and chronic optic neuropathy while showing neurotoxicity (Block et al., 2007; Fatteh et al., 2011; Subhramanyam et al., 2019) and promoting scar formation at the injured site (Jin & Yamashita, 2016). Tacrolimus (also known as FK506), a hydrophobic macrolide antibiotic, exert neuroprotective and neuro-regenerative effects following axon injury through inhibition of the over-activation of the immune response (Zawadzka et al., 2012). It also can reduce scar formation through induction of fibroblast apoptosis post nerve injury (Que et al., 2012, 2013).

Thus, we presumed that localized co-delivery of FK506 and CNTF *in situ* may suppress microglia over activation, restore the neurotrophins supply and can effectively protect RGCs post TON. Considering the anatomical limitation of optic nerve, a thermo-sensitive hydrogel was designed that can undergo sol-to-gel transition at the site of injury under physiological temperature hence may act as drug depot to sustainably supply the therapeutic agents is a preferential strategy for post TON RGCs protection. Chitosan (CS)based thermo-sensitive hydrogel, can undergo sol-to-gel transition upon increase in physiological pH and temperature due to its intrinsic properties including non-toxicity, biocompatibility, biodegradability, bioadhesive, and penetration enhancer properties, thus, CS-based thermo-sensitive hydrogel represents a suitable material to confers these purposes (Bhattarai et al., 2010; Dudhani & Kosaraju, 2010). However, the FK506 was difficult to encapsulate into a hydrogel due to its hydrophobic nature. Therefore, it was first loaded in an amphiphilic polymeric micelle. Subsequently, the FK506 loaded micelle and CNTF were co-encapsulated into the hydrogel after complete mixing with the base materials and then gelled at a physiological pH of 37 °C. The physicochemical properties of the hydrogel were characterized. And RGCs protective effects of drug-loaded hydrogel post TON were evaluated in an animal model.

## 2. Methods and materials

### 2.1. Materials

The γ-benzyl-L glutamate N-carboxy anhydride and *β*-sodium glycerophosphate (β-GP) were purchased from Aladdin Reagents (Shanghai, China). The methyl amino-poly (ethylene glycol; mPEG–NH2; Mw = 5000 Da) was provided by YareBio Co. (Shanghai, China). CS (120 kDa) and dehydrated N, N- dimethylformamide (DMF) were procured from Sigma-Aldrich (St. Louis, MO). The S/D rat originated primary retinal ganglion cells (RGC) was obtained from Procell Life Science and Technology Co., Ltd. (Wuhan, China). All reagents were of HPLC grade. Other chemicals and reagents were of analytical grade, obtained commercially.

### 2.2. Preparation and characterization of FK506 loaded nano-micelle

The amphiphilic methoxyl polyethylene glycol-block-poly (benzyl glutamate) copolymer (PEG–PBG) was synthesized (Luo et al., 2018). The FK506 loaded nano-micelle was prepared with dialysis as described previously (Lin et al., 2019), with minor modification. Briefly, 8 mg of FK506 and 32 mg of PEG-PBG were mixed and dissolved in 2 mL of N, N-dimethyl formamide (DMF) and was subjected to dialysis (MWCO 3500 Da) for 4 h against excessive distilled water allowing the formation of FK506 loaded nano-micelle by self-assembly. The micelle was sterilized by UV-exposure for 3 h. The size and zeta potential of nano-micelle were measured using dynamic light scattering (DLS) with a ZS-90 zeta-sizer (Malvern, UK). To determine the FK506 loading amount, the micelle was freeze-dried and re-dissolved in DMF to achieve a final concentration of 1 mg/mL (FK506 and PEG–PBG in DMF). After appropriate dilution, it was analyzed quantitatively with a Shimadzu LC-8050 UPLC-MS-MS system according to the procedures described previously (Lin et al., 2019).

### 2.3. Preparation of thermo-responsive hydrogel

A CS-based thermo-responsive hydrogel was prepared using β-GP as the gelatin agents with the methods described by Deng et al (2017), with slight modification. Briefly, CS was dissolved in 0.1 mol/L acetic acid. The *β*-sodium glycerophosphate (0.7 g/mL in distilled water) was added to the solution dropwise over 10 min with magnetic stirring under an ice bath to achieve the final pH of 7.2. After homogenous mixing, the hydrogel was formed when the temperature escalated to 37 °C. To prepare drug-loaded hydrogel, the FK506 micelle (17.6 μg) and CNTF (20 μg) were suspended/dissolved in 10 mL CS solution (in 0.1 mol/L acetic acid). It was gelled using the above-mentioned method.

### 2.4. Rheologic characterization

The rheological assessment of the hydrogels was performed as described previously (Wang et al., 2018), with slight modification. The experiments were performed on a rheometer (AR-2000, TA Instruments, USA) in oscillatory mode configurated with a 40 mm (diameter) parallel plate. The gap between the parallel plates was set at 300 μm. To determine the heat triggered gelling property of hydrogel, the storage modulus (G′) and loss modulus (G″) changes were determined within the temperature range of 4–45 °C with the following process: the hydrogel was initially soaked in 4 °C for 2 min, and temperature was escalated to 45 °C at a speed of 1 °C/min. To measure the gel time at physiological condition, G′ and G″ were recorded at 37 °C with a function of time under a constant frequency of 1 Hz and a constant strain of 1%. The gelation point was considered to be the point at which the loss and storage moduli values were the same.

### 2.5. Morphologic characterization

After gelling at 37 °C, the hydrogel was snap-frozen in liquid nitrogen for 15 min. After freeze-dried, a small piece of the dried material was acquired manually and fixed on electroconductive paste. The microstructure of the hydrogel was determined on a Phenom LE scan scanning electron microscope (SEM, PhenomScientific, Netherlands).

### 2.6. *In vitro* drug release

For drug release studies, the FK506 release profile from micelles was evaluated using the method described in our previous report (Lin et al., 2015), with slight modification. Briefly, 1 mL of FK506 loaded micelle suspension was enclosed in a dialysis bag (MWCO 3.5 kDa), and immersed in a tube containing 20 mL of distilled water. The tubes were then treated under 37 °C in a rotation shaker with a rotation speed of 150 rpm. At the designated time interval, 1 mL of dialysate was sampled and the sampled volume was replaced with fresh solution. The samples were freeze-dried and re-dissolved in methanol. The concentration FK506 was measured by UPLCMS-MS. Furthermore, the FK506 release profile was determined after the FK506 loaded nano-micelle embedded into the hydrogel using the method as described earlier, with an exception that FK506 loaded hydrogel was used instead of the micelle.

The protein release from the hydrogel was analyzed using FITC-BSA as the model drug. FITC-BSA was encapsulated into the hydrogel using the method described in section 2.4. A mixture of CS (1%; 3 mL) and fluorescein isothiocyanate labeled bull serum albumin (FITC-BSA;1.5 mg) and FK506 nanoparticle (contains 26.4 μg FK506) was prepared in a 15 mL glass chamber. After adjusting the pH value to 7.2 with β-GP, the mixture was gelled at 37 °C. The surface of the gel was gently washed with PBS (37 °C), an additional 3 mL of PBS was added as release matrix. At the designated time interval, 1 mL of the release matrix was sampled and replaced with the same amount of fresh matrix. The released FITC-BSA was quantified by fluorescence intensity at 550 nm with the excitation at 493 nm. The amount of FITC-BSA was quantified by comparing it with the corresponding standard curve.

### 2.7. Cytotoxicity evaluation

The cytotoxicity of hydrogel against RGC was evaluated using a Cell Counting Kit-8 (CCK-8) assay (Dojindo, Japan). The primary RGCs from Sprague-Dawley (SD) rats were kindly provided by Procell life science and technology Co. LTD, Wuhan, China. Rat RGCs were cultured in Neurobasal-A/B27 medium containing 2% B-27 as a supplement in a humidified atmosphere of 5% CO_2_ and 90% relative humidity at 37 °C. Briefly, RGCs suspension was seeded in a 24-well plate at a density of 4 × 104 per well. After 24 h of incubation, the trans-well chamber with 8 μm pore was used for substance exchange containing different amounts of hydrogel hanged above the cell layer. At designated time point, the culture medium was replaced with PBS supplemented with 10% of CCK-8 solution. The cell viability was determined by measuring the absorbance at a wavelength of 450 nm with a microplate reader (Bio-Rad, USA).

### 2.8. *In vivo* post TON RGC protective effects

All the animal experiments were approved by the Animal Ethics Committee of the Ethical Review Committee for Experimental Animals of Wenzhou Medical University (WMU), China. Mice were cared for in accordance with the National Institute of Health (NIH) Guide for the Care and Use of Laboratory Animals.

Nine healthy Japanese white rabbits (male, 2.0 ± 0.2 kg in weight, 3–4 months old) were procured from Jiesijie experimental animal CO. (Shanghai, China). Animals were anesthetized and the optic nerve was exposed by surgical procedure. The TON animal model was prepared by clamping the optic nerve (2 mm postocular) for 30 s with reverse artery-clamp. A liquid (200 μL) containing 1% of CS, 1.76 μg/mL of FK506 (in micelle) (Zhao et al., 2014), 2 μg/mL of CNTF (Laughter et al., 2018), and β-GP was smeared at the injured site of optic nerve, and gelled under physiology temperature forming a sheet acting as a *in situ* drug depot for the sustainable drug release at the injured site of optic nerve. The retina, optic nerve, and RGC was evaluated on the 14^th^ day after the surgical procedure.

On 14-day post TON, the rabbits were sacrificed by injecting air at ear vein. The eyeball was removed immediately and fixed in 4% paraformaldehyde at 4 °C for 48 h and transferred sequentially into 5% and 30% saccharose solution, each at 4 °C overnight. Subsequently, the tissues were soaked in Optimum Cutting Temperature (OCT) media, and frozen in liquid nitrogen immediately. Eye sagittal cryosections (14 μm) were obtained with a cryostat and mounted on gelatin-coated slides. The cell apoptosis was evaluated using terminal deoxynucleotidyl transferase-mediated dUTP nick end labeling (TUNEL) staining kit (Roche, Mannhein, Germany) according to the manufacturer’s protocol. The fluorescence images were obtained with a Zeiss confocal microscope (Germany) at an excitation wavelength of 405 nm and 488 nm for DAPI and TUNEL, respectively. To assess the *in vivo* immune response after TON, the sections were incubated with anti-ionized calcium binding adapter 1 molecule (anti-Iba-1, 1:100, Abcam, ab178846) and Cy3 donkey anti-Rabbit (1:500, Jackson lab) in sequence. The paraffin-embedded section (5-μm) were then prepared and subjected to hematoxylin and eosin (H&E) staining.

### 2.9. Statistical analysis

The data was analyzed by OriginPro 8 (OriginLab CO., Massachusetts, USA). The one-way analysis of variance (ANOVA) followed by the Tukey − Kramer test was performed to analyze significant differences among the groups. The value of *p* < .05 was considered statistically significant.

## 3. Results

### 3.1. Characterization of FK506 loaded nanoparticle

**Figure 1.**
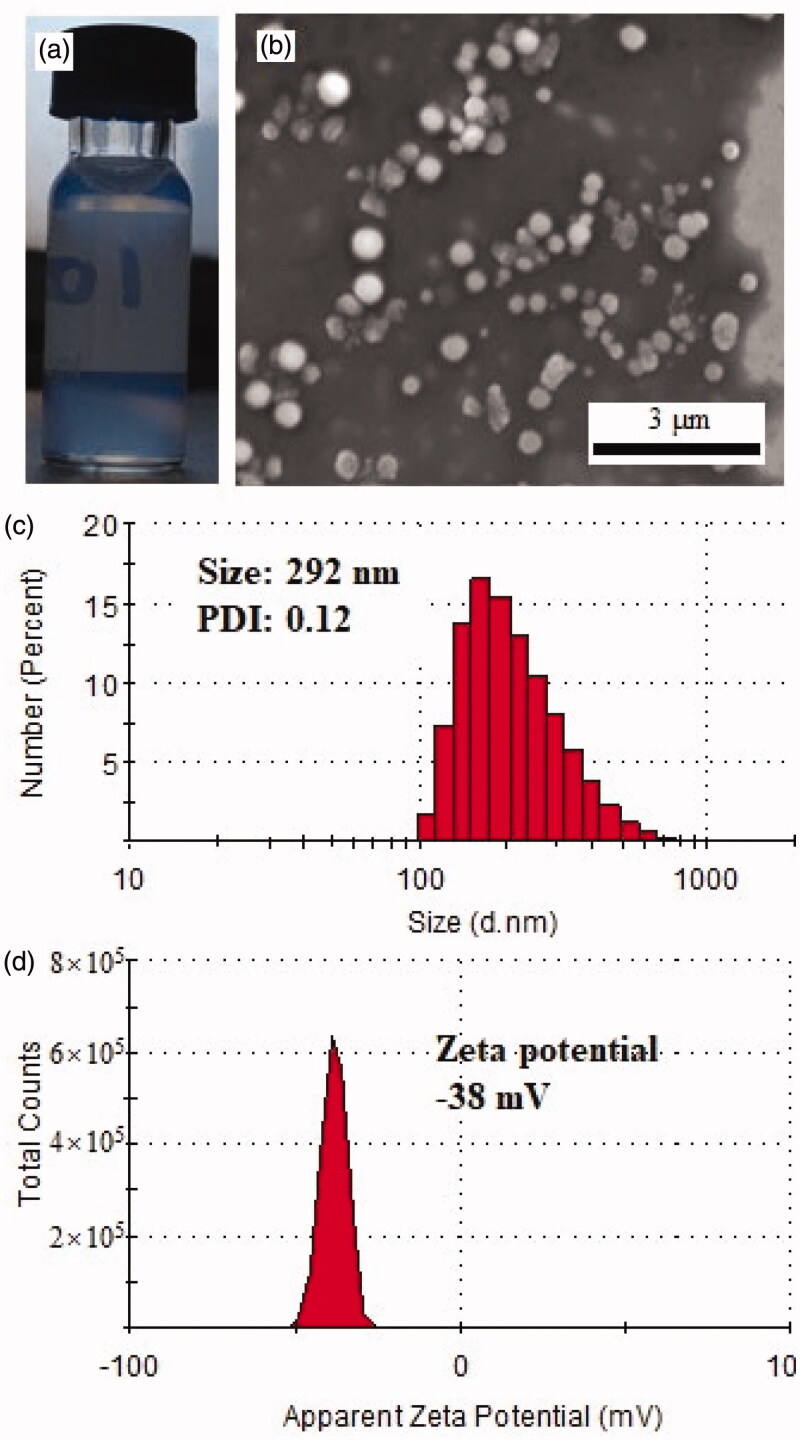
Formation of FK506 loaded micelle. (a) the representative image of FK506 loaded micelle; (b) the SEM image of FK506 loaded micelle; (c) the hydrodynamic diameter size distribution of FK506 loaded micelle; (d) the zeta potential of FK506 loaded micelle.

The PEG-PBG co-polymer was synthesized using the ring-opening polymerization method and characterized with ^1^H Nuclear magnetic resonance (^1^H-NMR) and Fourier transform infrared spectroscopy (FTIR) as reported previously (Luo et al., 2018). It exhibited characteristic optical properties after self-assembly forming micelle (Figure 1(a)). After co-self-assembled with FK506 by dialyzing, this micelle exhibited a loading capacity of FK506 of 11%, which was relatively close to a previously prepared peptide-decorated PEG-PBG micelle (Lin et al., 2019). It formed a nanosphere with a size range from 100–350 nm as observed with SEM (Figure 1(b)). These findings were consistent to some extent echoed with the results of DLS that revealed a hydration size distribution of 150–500 nm (peak size of 303 nm) and a PDI of 0.12 (Figure 1(c)). Generally, the particle size measured by DLS represents the hydration size larger than observed by SEM (dry size), which was also observed in other nano-particles with different materials (Lin et al., 2015; Yuan et al., 2015; Yang et al., 2017). This FK506 loaded nanoparticle had a zeta potential of −38 mV (Figure 1(d)), indicating stability the nano-formulation for biological application (Ostolska & Wiśniewska, 2014).

### 3.2. Characterization of hydrogel

**Figure 2.**
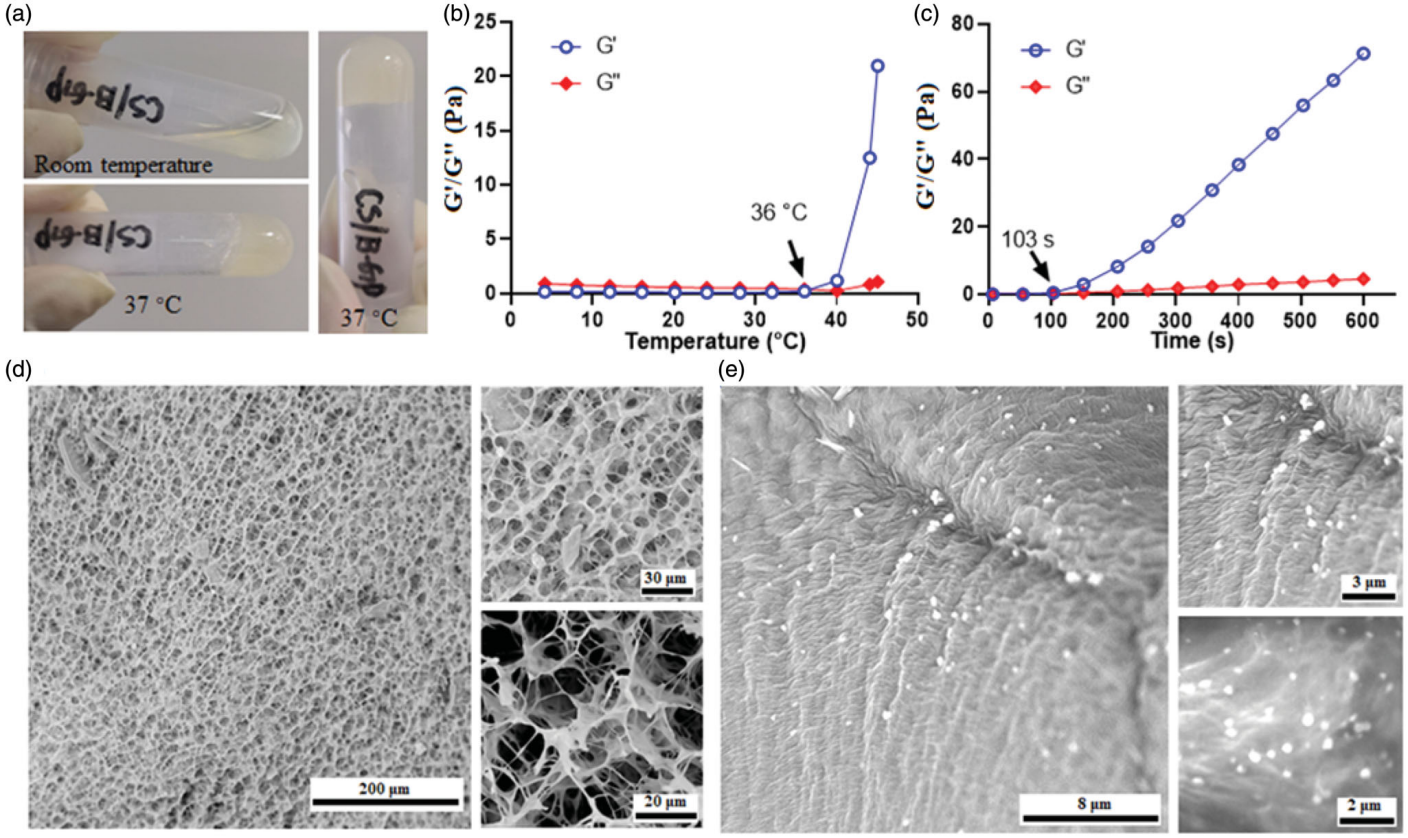
Formation of the CS based hydrogel. (a) the representative images of thermosensitive hydrogel; (b) the temperature dependent changes of modulus of the hydrogel; (c) the time dependent changes of modulus of the hydrogel; (d) the representative SEM images of the porous hydrogel; (e) the representative SEM images of the micelle in the hydrogel.

The mixture containing CS (1%) and gelling agent (β-GP) presented in a liquid state under room temperature. However, it was gelled when the temperature was raised to 37 °C (Figure 2(a)). The modulus change during the gelling process was recorded by a rheometer. As presented in Figure 2(b), the storage modulus (G′) maintained at a low value and exhibited no apparent difference with the loss modulus (G″), when the temperature was set below 36 °C. A dramatic change in storage modulus was observed when the temperature was increased from 36 to 45 °C, indicating the formation of hydrogel (Deng et al., 2017). In order to understand how fast it will be gelled, the modulus changes below 37 °C were monitored as a function of time. As shown, the mixture starts gelling after 37 °C treatment for 103 s (Figure 2(c)), leading to an increase of G′. The mechanism underlies this sol-to-gel transition mainly attributed to heat causing the enhancement of the electrostatic attractions of CS and β-GP; besides, heat-induced the hydrophobic interactions between CS molecules resulted from the reduction of its chain polarity, and heat triggered protons transfer from CS to β-GP led to the reduction of interchain electrostatic repulsion in CS molecule (Zhou et al., 2015; Deng et al., 2017). The morphology of the hydrogel was observed with SEM after quick-freeze in liquid nitrogen. As illustrated in Figure 2(d), it exhibited a porous structure with a pore size of 10–20 μm. Upon gelling with FK506 loaded micelle, particles with a size around 300 nm were observed inside the pore, indicating successful encapsulation of the micelle into a hydrogel (Figure 2(e)).

### 3.3. *In vitro* drug release profile

**Figure 3.**
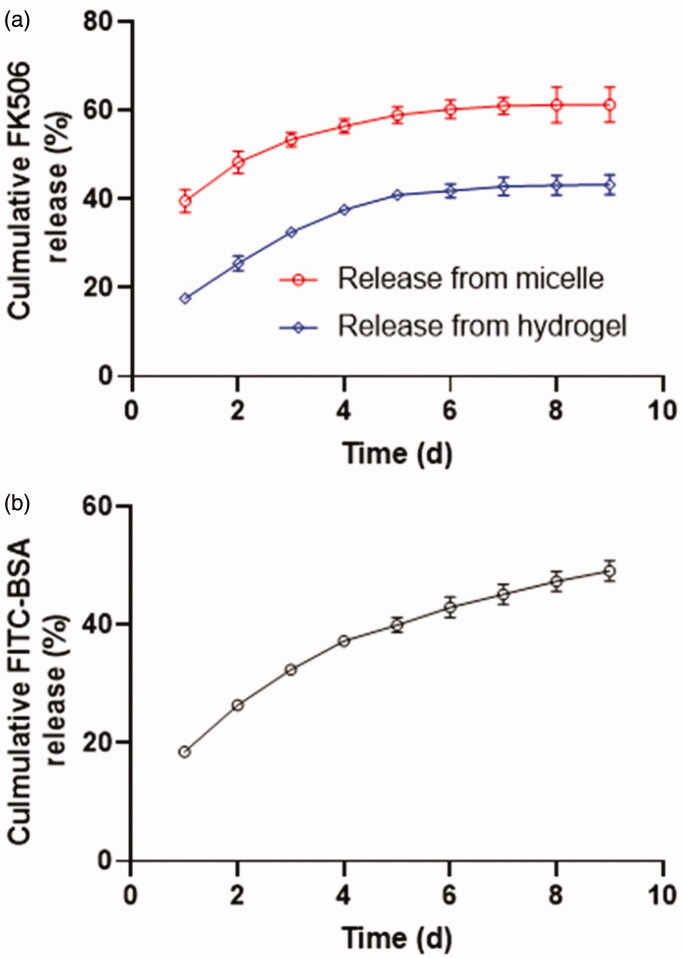
the invitro release profile of FK506 (a) and the FITC-BSA (b).

For the drug release study, the FK506 was first encapsulated into a polymeric micelle and subsequently incorporated into CS-based hydrogel. Figure 3(a) depicted the release profile of FK506 from the micelle. The FK506 presented a sustainable release profile with 60% of the drug was released from micelle in 9 days following surgical procedure, which was relatively close to a previously prepared peptide-decorated PEG-PBG micelle (Lin et al., 2019). However, the release rate was lowered when this FK506 loaded micelle was introduced into the CS-based hydrogel. After an incubation for one day, around 20% percent of FK506 was released from hydrogel compared 40% of drug was released from micelle, possibly due to the non-covalent interactions between FK506 and CS skeleton (compose of β-(1–4)-linked D-glucosamine and N-acetyl-D-glucosamine) and the retarded effect of network of hydrogel for FK506 diffusion. Moreover, in total 40% of FK506 was released from the hydrogel in 9 days. The release profile of the protein drug from the hydrogel was determined using FITC-BSA as the fluorescent probe. As presented in Figure 3(b), approximately 20% of FITC-BSA was released from the hydrogel and the cumulative release amount reached 60% in duration of 9 days.

### 3.4. Cytotoxicity

**Figure 4.**
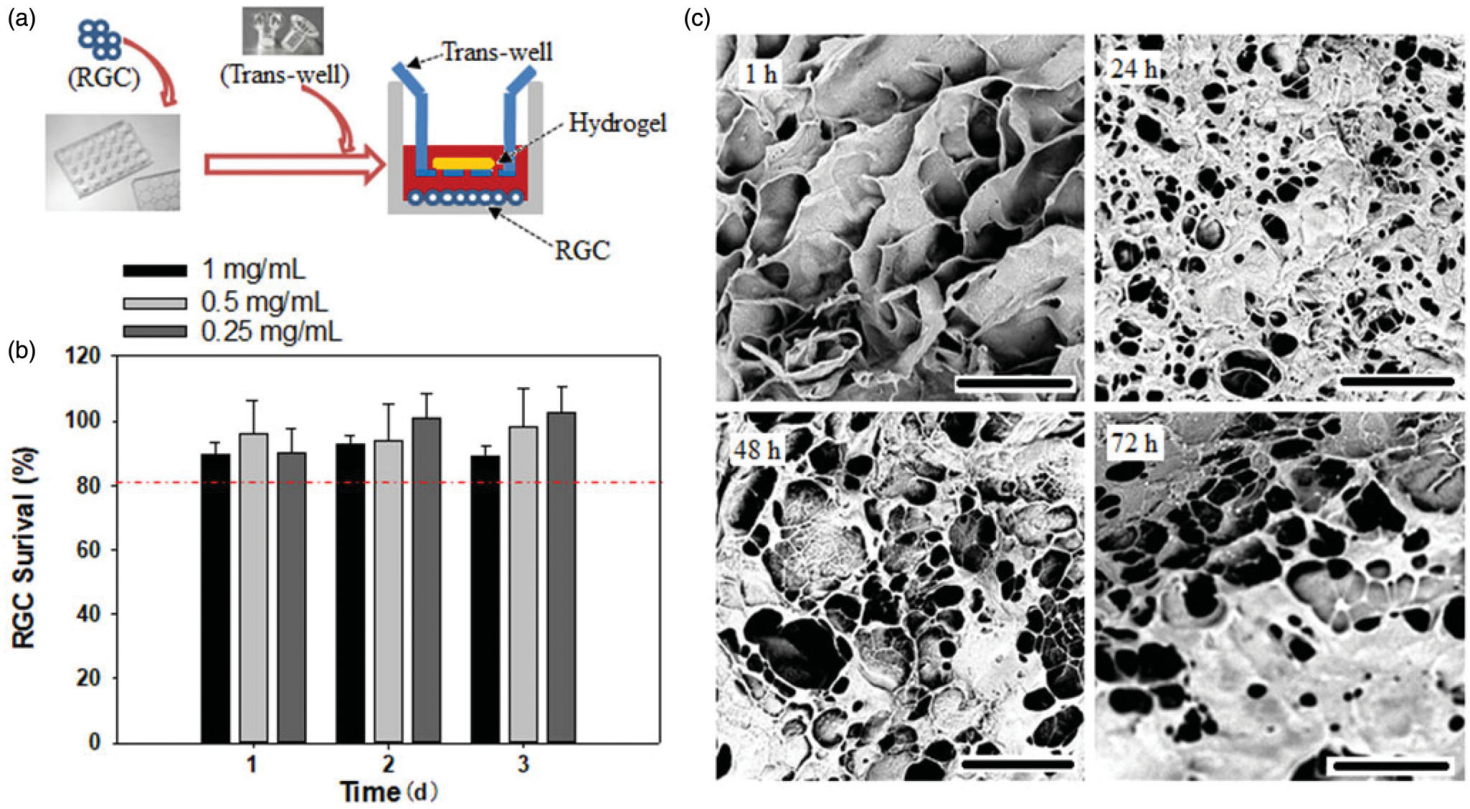
The cytotoxicity evaluation of the bank hydrogel (a and b) and the morphologic changes of hydrogel over time by co-culture with RGCs (c).

The cytotoxicity of the hydrogel against RGCs were evaluated using a CCK-8 assay. As presented in Figure 4(a,b), over 80% of RGCs survived within 3 days of culture; however, no significant difference in the survival rate was observed among the hydrogel concentration of 0.25, 0.5, and 1 mg/mL, suggesting this CS-based hydrogel exhibited good cell compatibility, which was consistent with previous finding on corneal endothelial cells and L929 mouse fibroblast cell line (Zhou et al., 2015; Kong et al., 2017). The morphologic alteration of the hydrogel after incubation with RGCs for different time periods was observed with SEM. As shown in Figure 4(c), most of the pore structure of the hydrogel was retained for 72 h on co-culturing with RGCs, indicating that this hydrogel existed for >72 h, possibly due to the resistance of CS against biodegradation (Kean & Thanou, 2010), suggesting its suitability for application as a localized drug depot.

### 3.5. *In vivo* RGCs protective effects

**Figure 5.**
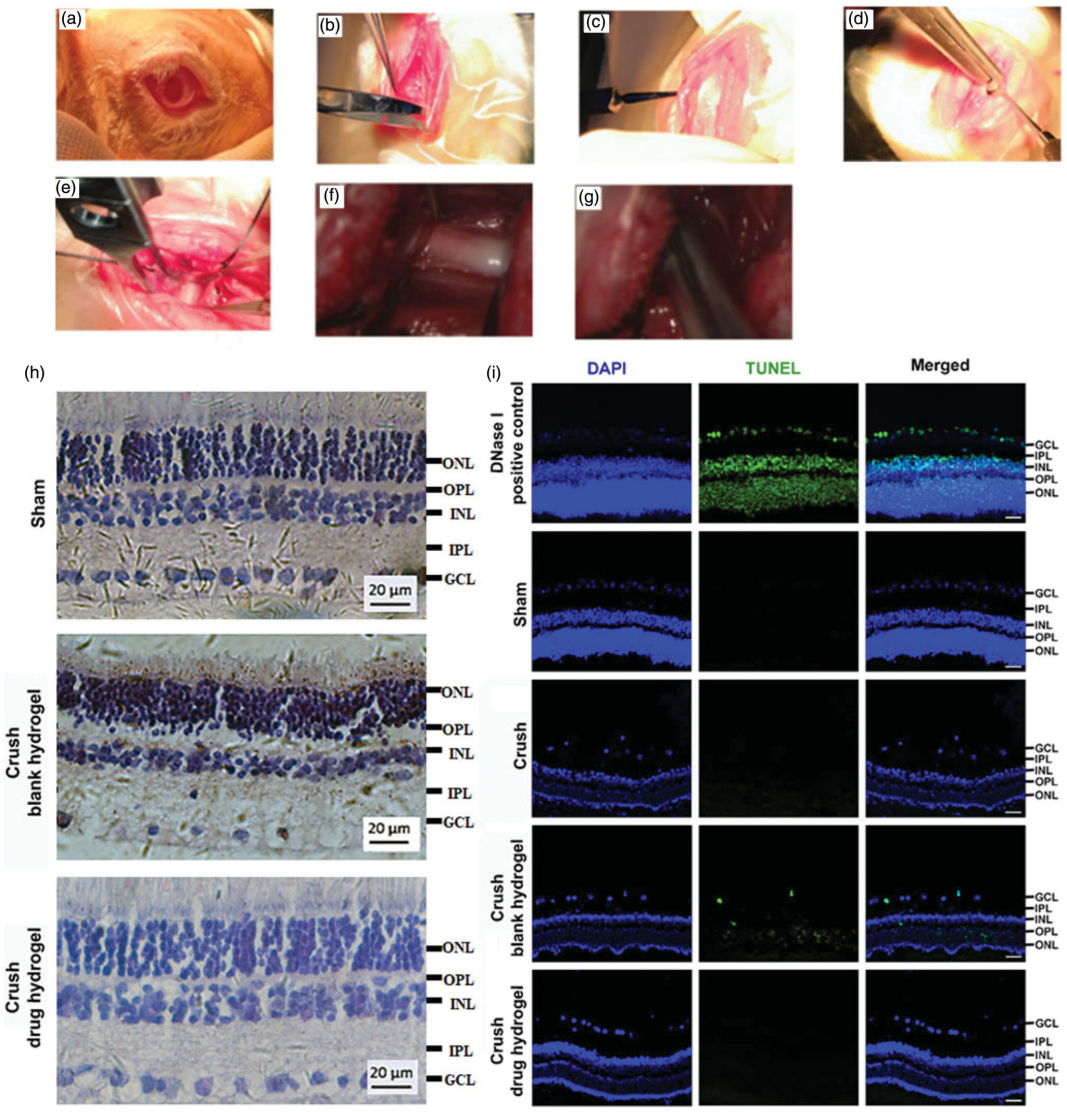
The surgical procedure to expose the optic nerve (a-f) and crush of optic nerve by reverse artery clamp (g). The H&E (h) and TUNEL (i, scale bar 20 μm) staining of retina from rabbits.

**Figure 6.**
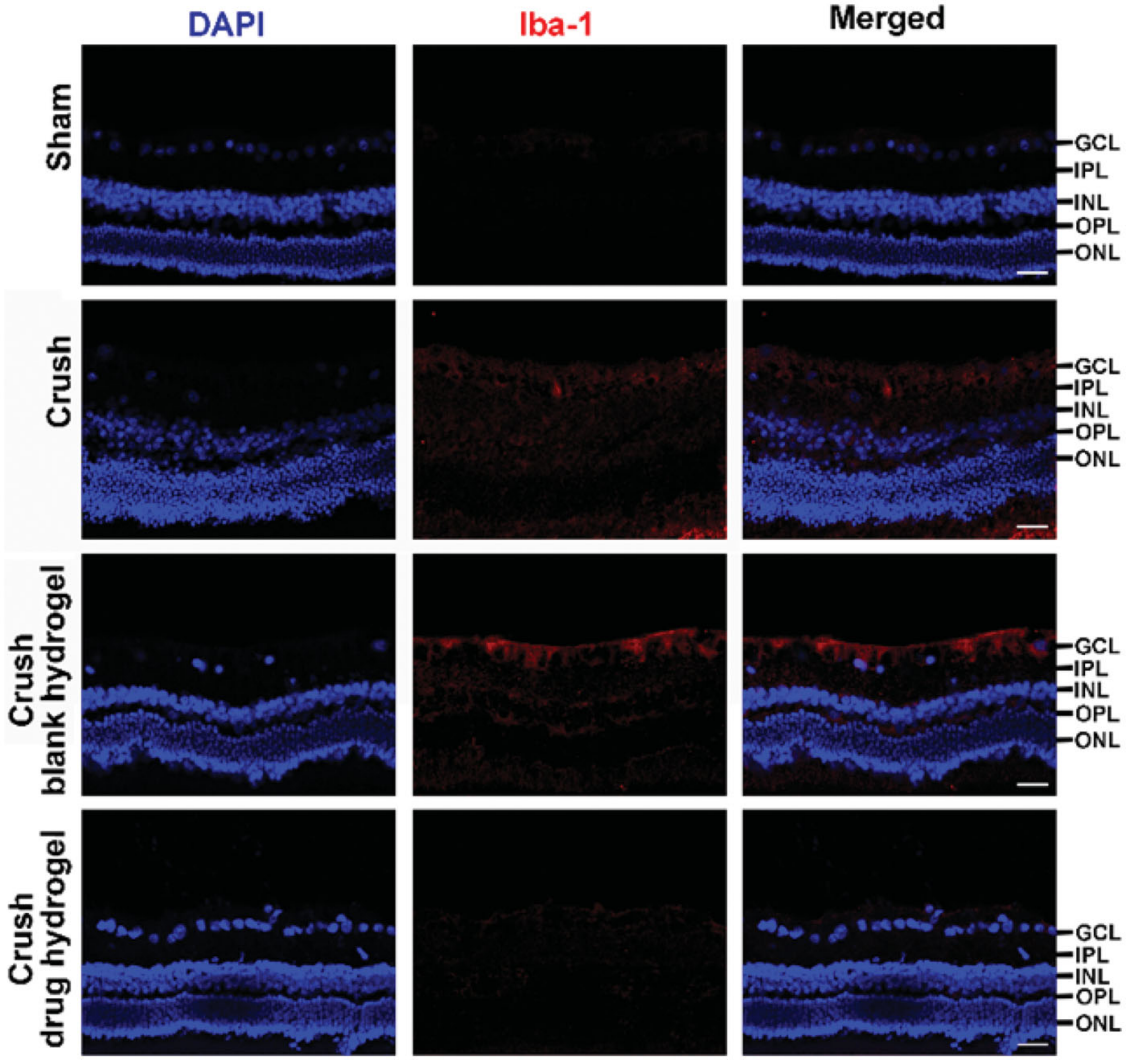
The Iba-1 expression profiles in retina from rabbits after receiving different treatment. Scale bar =20 μm.

The animal model for TON was induced on Japanese white rabbits. The optic nerve was exposed by surgical procedure (Figure 5(a–f)) and was crushed by a reverse artery clamp (Figure 5(g)). A mixture containing CS, FK506 (in micelle), CNTF, and the gelling agent (β-GP) was smeared at the injured site (and nearby) of optic nerve. To improve the drug penetration, the sheath membrane of the optic nerve was gashed along the nerve fibers. After it gelled, the wound was sutured. The experimental animals were sacrificed and the sections of the retinal were prepared. The RGCs protective effects of the drug-loaded hydrogel was evaluated based on H&E staining as well as the TUNEL analysis. As illustrated in Figure 5(h), the retina from healthy rabbits exhibited characteristic ordered structure comprised of ganglion cell layer (GCL), inner plexiform layer (IPL), inner nuclear layer (INL), outer plexiform layer (OPL), and outer nuclear layer (ONL), (Bringmann & Reichenbach, 2009). Following injury, the tissue of GCL exhibited incomplete morphological structures and a large proportion of RGCs was missing, indicative caused the damage of the RGCs. However, very few TUNEL positive cell was observed, probably because the dominating pathway of RGCs death following TON were not via cell apoptosis. Our unpublished data suggested that ‘ferroptosis’ can play an important role in RGCs death following TON. however more efforts are need to clarify the mechanism underlie. After co-treating with FK506 and CNTF containing hydrogel, the GCL from crashed animals became more regular in morphology and fewer RGCs death was observed (Figure 5(h,i)), suggesting this hydrogel exhibited a potential to protect RGCs after axonal damage. The Iba-1 staining was conducted to illustrate the immune responses after receiving different treatment. As shown in Figure 6, decreasing in Iba-1 positive cells was observed after co-treating with FK506 and CNTF containing hydrogel, suggesting this co-delivery system can suppress the microglial activation following TON.

## 4. Discussions

The localized drug delivery can achieve 100-fold or higher concentration of drug at the focal site than systemic administration, which substantially enhances the therapeutic efficiency and reduce off-target side effect (Wolinsky et al., 2012). CS has a lengthy history of application and investigation as a biosafety-material for extensive biomedical applications (Kean & Thanou, 2010). It was developed for obtaining hydrogel by physical association or chemical cross-linkage for localized drug delivery (Bhattarai et al., 2010). The injectable thermosensitive hydrogels with a lower sol-gel transition temperature around physiological temperature are most suitable for localized drug delivery as *in situ* drug depot with the additional advantage of the pre-engineered hydrogel that can locally deliver bioactive drugs thereby circumventing invasive surgeries (Bhattarai et al., 2010). Therefore, in this study, we have developed a CS-based thermo-sensitive hydrogel formed by the interaction of CS and β-GP. The pre-hydrogel solution gelled in minutes under the physiological temperature of 37 °C, generated a porous network structure and exhibited good compatibility with RGCs. Notably, triggered by heat, this CS-based network was generated by comprehensive effects of improved electrostatic attractions between CS and β-GP, reduced CS inter chain electrostatic repulsion, and enhanced hydrophobic interactions between CS molecules resulted from the reduction of its chain polarity (Zhou et al., 2015; Deng et al., 2017).

Sustainable supplies of neurotrophins are essential for RGCs survival as well as for maintaining its physiological function. Deprivation of neurotrophins in RGCs and the overactive immune responses together constitute the predominant cause for RGCs death following optic nerve injury. The present study presented a CNTF and FK506 co-encapsulated hydrogel for localized co-delivery of these two therapeutic agents to restore neurotrophins supply concurrently suppressing the overactive immune responses following traumatic optic nerve injury. Hydrogels are characterized as a high-water content material, which are difficult to load with hydrophobic molecule (Bhattarai et al., 2010). However, with appropriate modification producing hydrophobic domains, they can be tailored to encapsulate hydrophobic molecules effectively (Bhattarai et al., 2010; Lin et al., 2018). In this study, we first encapsulated hydrophobic molecule (FK506) into a polymeric micelle safeguarding a hydrophilic outer sphere; subsequently, loaded into the hydrogel for a desirable drug distribution inside the hydrogel. Then, *in vivo* RGCs protective effects of this co-delivery system were evaluated on an *in vivo* optic nerve injury model of the rabbit. Results indicated this strategy exhibited RGCs protective effect against the adverse circumstances instigated by traumatic optic nerve injury, indicating the potential of this drug delivery system for effective optic nerve repairs.

The focal of traumatic optic nerve injury is usually located at the back of the eyeball, making the treatment even more challenging for localized drug delivery owing to the anatomical limitation. Therefore, we have developed a strategy to deliver therapeutic agents directly to the back of eyeball through the nose under guidance of trans-nasal endoscope in big experimental animals (unpublished data). Apparently, it is warranted to screen constituents and drugs in small experimental animals to achieve the desirable bio-effect *in vivo*. However, the channel in small animals (rabbits) from the nose to the back of the eyeball is too narrow to perform the trans-nasal endoscope procedure. Therefore, in this study, we exposed the optic nerve of rabbits through surgical procedure and directly smeared the drug-loaded hydrogel on the focal to evaluate the bio-effects of this material with the less challenging approach. Based on these findings on rabbits, we look forward to reproducing it on large animals for improved localized delivery of therapeutic molecules using a less invasive approach with the help of a trans-nasal endoscope.

## 5. Conclusions

The present study developed and designed a CS-based thermosensitive hydrogel for localized and sustainable co-delivery of CNTF and FK506 to the optic nerve. The as-synthesized hydrogel exhibited a uniform porous network structure and showed good compatibility with RGCs. The drugs were sustainably released for a period of 9 days *in vitro*. This co-delivery system exhibited *in vivo* RGCs protective effect against the adverse effects caused by traumatic optic nerve injury, indicating the potential of this drug delivery system for effective optic nerve repair and this strategy may provide promising platforms for localized drug delivery in various other therapies.
